# Greater Pain Severity is Associated with Higher Glucocorticoid Levels in Hair Among a Cohort of People Living with HIV

**DOI:** 10.2147/JPR.S301651

**Published:** 2021-03-09

**Authors:** Quan Zhang, Xiaoming Li, Shan Qiao, Shuaifeng Liu, Zhiyong Shen, Yuejiao Zhou

**Affiliations:** 1 South Carolina SmartState Center for Healthcare Quality (CHQ), Arnold School of Public Health, University of South Carolina, Columbia, SC, USA; 2 Institute of Pedagogy and Applied Psychology, School of Public Administration, Hohai University, Nanjing, Jiangsu, People’s Republic of China; 3 Guangxi Zhuang Autonomous Region Center for Disease Control and Prevention, Nanning, Guangxi, People’s Republic of China

**Keywords:** pain, hair cortisol, hair cortisone, HIV

## Abstract

**Background:**

Pain is a common occurrence and persistent symptom, which has an adverse impact on individual well-being and quality of life among people living with HIV (PLHIV). Alteration in the activity of the Hypothalamic-Pituitary-Adrenal (HPA) axis resulting in abnormal glucocorticoid levels had been proposed to play important roles in those associations.

**Purpose:**

This study aimed to investigate whether pain severity was associated with hair glucocorticoid levels, a novel method of measuring long-term glucocorticoid exposure, among a large cohort of Chinese PLHIV.

**Methods:**

A measure of pain severity and hair samples were collected from 431 adults PLHIV in Guangxi, China. Glucocorticoid (cortisol and cortisone) in hair were quantified by liquid chromatography-tandem mass spectrometry. The general linear model was used to test the associations of pain severity with hair glucocorticoid levels after adjusting for potential confounding factors.

**Results:**

Of the 431 PLHIV, 273 reported none pain, 87 reported mild pain, and 71 reported moderate-severe pain. Hair cortisone, but not hair cortisol, was found to differ significantly among the three pain severity groups (*F*=3.90, *p*=0.021). PLHIV reported moderate-severe pain had higher hair cortisone than those reported mild (*p*=0.070) or none pain (*p*=0.014), with no differences between the latter two pain severity groups.

**Conclusion:**

Greater pain severity is associated with higher hair cortisone levels among Chinese PLHIV. In order to reduce the long-term glucocorticoid levels, interventions managing pain should be considered for PLHIV with moderate-severe pain.

## Introduction

Pain is one of the most commonly reported and persistent symptoms among people living with HIV (PLHIV), affecting 54% to 83% PLHIV, as indicated by a systematic review.^1^ The etiology of pain in PLHIV is varied and includes the direct effects of HIV on nervous systems, resultant opportunistic infections, side effects of combination antiretroviral therapy (cART), or other conditions unrelated to HIV.^2^ Regardless of etiology, pain in PLHIV is a significant source of function loss^3^ and decreased quality of life,^4^ and has been associated with elevated levels of psychological distress, including anxiety and depression,^5–8^ as well as higher adverse behaviors, including substance use^9^,^10^ and suboptimal ART adherence.^11–13^ While the increased attention has been paid to the relationship between pain and these adverse outcomes in PLHIV, limited data are available concerning the biological mechanisms underlying these associations in PLHIV.

The hypothalamic-pituitary-adrenal (HPA) axis is one of the main stress-sensitive systems regulating the body’s adaptation to stress by secretion of the glucocorticoid hormone.^14^,^15^ There is substantial evidence for a relation of hyper- or hypo-secretion of glucocorticoid with adverse health outcomes.^16^ Pain has been conceptualized as one type of stress that adds strain on the organism.^17^,^18^ Therefore, glucocorticoid is considered to play a crucial role in mediating the link between pain and the development of adverse health outcomes. While previous studies have investigated the relationship between pain and glucocorticoid levels in patients with chronic pain,^19–24^ critical gaps in the relationship between pain and glucocorticoid levels in PLHIV remain unaddressed.

As well known, cortisol is an active glucocorticoid, and cortisone is an inactive glucocorticoid originating from the local conversion of cortisol by the 11β hydroxysteroid dehydrogenase (11β-HSD) type 2 enzyme, and 11β-HSD type1 enzyme is responsible for the reversible conversion of cortisone to cortisol.^25^ Therefore, the interaction between cortisol and cortisone regulates stress-induced psychological and physiological responses together.^26^,^27^ In addition, cortisone levels are higher than those of cortisol levels in biomatrix (e.g., saliva and urine).^25^,^28^ Moreover, cortisone levels could more closely approximate unbound, biologically active cortisol levels than total cortisol levels.^25^ Therefore, the assessment of cortisone in parallel with cortisol could provide a more systematic evaluation of the glucocorticoid exposure.^29^

The conventional methods for cortisol and cortisone assessment in serum, saliva, or urine are susceptible to reflect mainly short-term cortisol and cortisone levels and affect by the circadian rhythm and other daily fluctuations.^30^ Recently, another method measures cortisol and cortisone levels in scalp hair, which circumvents many limitations of the previous methods and enables retrospective assessment of cortisol and cortisone levels in the past weeks to months, depending on the length of the collected sample.^31^ Comparing between hair cortisol and cortisone levels and cortisol and cortisone levels in repeated saliva sampling,^29^,^32–34^ testing the test-retest reliability of hair cortisol and cortisone levels across a period of several months to a year,^28^,^35^ and employing hair cortisol and cortisone levels in stress or chronic disease research,^36–40^ all suggest that hair cortisol and cortisone levels are novel retrospective indicators of long-term glucocorticoid exposure. To our knowledge, only two studies employed hair cortisol levels in HIV-related research.^41^,^42^ Limited data are available on employing hair cortisone levels in HIV-related research, and no study examined the association between pain severity and glucocorticoid levels in PLHIV by employing hair cortisol and cortisone levels.

Accordingly, we assessed pain severity and hair cortisol and cortisone levels in a large cohort of PLHIV in Guangxi, China, and aimed to examine whether pain severity was associated with hair cortisol and cortisone levels among Chinese PLHIV.

## Methods

### Participants

The participants of this study were recruited from an HIV disclosure study aiming to investigate the mechanism of the effects of HIV disclosure on clinical outcomes in Guangxi, China.^43–45^ With the assistance and collaboration of the Guangxi Center for Disease Prevention and Control (CDC), ten clinic sites with the largest number of HIV patients under care from 17 cities and 75 counties in Guangxi were selected as study sites.

The inclusion criteria were: (1) at least 18 years of age; (2) a confirmed diagnosis of HIV; (3) willing to provide hair samples; and (4) willing to consent the retrieval of their relevant clinical outcome data (eg, CD4+ T cell count) from their medical charts. The exclusion criteria were: (1) linguistic, mental or physical inability to respond to assessment questions; (2) opportunistic infections, coinfection, comorbidities, endocrine diseases, or reported any other diseases; (3) currently take hormonal drugs (eg, prednisolone); (4) known history of drug use; (5) chemical hair treated (eg, dyed, permed, or bleached) or scalp hair in the posterior vertex was less than 1 cm.

Medical staff or HIV case managers at the study sites referred potential participants to the research term members. Research team members screened PLHIV for eligibility, discussed the benefits and risks of the study, and invited them to participate. Research team members were local CDC staff or health care workers in the HIV clinics who had received intensive training on research ethics and interview skills with PLHIV before the field data and hair specimen collection. Finally, a total of 446 PLHIV participated in this study. This study followed the Declaration of Helsinki and was approved by the Institutional Review Boards of the University of South Carolina in the United States and the Guangxi CDC in China. The written informed consent also has been obtained from the participants.

### Hair and Data Collection

Hair samples were cut from the posterior vertex region as close as possible to the scalp following a standard protocol^46^,^47^ in private rooms of local CDC or HIV clinics. The hair strands were cut with iron scissors that had been wiped with an alcohol pad. The hair thatch then was completely enclosed by a piece of foil, and a small label indicating the study ID number was placed over the distal end of the hair thatch. Then, the interviewer-administered questionnaire was used for data collection. After the hair sample collection, the interviewer-administered questionnaire was used for the collection of sociodemographic, lifestyle, and HIV-related information. Each participant received a gift with a value equal to the US $5.00 (≈ 35 Chinese Yuan) after finished the survey.

### Pain Measure

To assess pain severity, we asked participants the following single-item question: “how much pain have you generally had during the past month”.^48^,^49^ Response options were on a 6-point scale ranging from “none” to “very severe.” Given that pain data are generally skewed, we divided our sample into three groups: those who reported none pain, those who reported mild pain, and those who reported moderate-severe pain.

### Hair Cortisol and Cortisone

The proximal 1 cm of hair segments (approximately reflect the last month of accumulative cortisol and cortisone levels) was cut finely with scissors, and 20 mg were processed and analyzed using liquid chromatography-tandem mass spectrometry (LC/MS/MS) followed the protocol described by Gao and colleagues.^50^ The method showed good linearity (R^2^ > 0.99) in the range of 0.25–1250 pg/mg for both cortisol and cortisone, the limit of detection (LOD) for both cortisol and cortisone at 0.1 pg/mg, and the limit of quantitation (LOQ) for both cortisol and cortisone at 0.3 pg/mg. Intra-day and inter-day percentages coefficient of variation (CV) were less than 7% at standard concentrations of 1.25, 25, and 250 pg/mg, and recovery ranged between 95% and 107% for both cortisol and cortisone.

### Sociodemographic and Clinical Characteristics

Participants provided information on their sociodemographic that might have a potential influence on hair cortisol and cortisone levels,^30^,^51^,^52^ including age (years), gender (male vs female), ethnicity (Han vs non-Han), marital status (married vs other), education level (> 9 years vs ≤ 9 years), employment status (employed vs unemployed), monthly household income level (≥ 3000 Yuan vs < 3000 Yuan).

Clinical characteristics were abstracted from the participants’ medical charts, including the date of HIV diagnosis, cART status, and CD4+ T cell count. Years of HIV diagnosis referred to the period from the initial date of confirmed HIV diagnosis to the time of the survey. CD4+ T cell count was dichotomized as >500 cells/mm^3^ vs ≤500 cells/mm^3^ because the low limit of normal CD4+ T cell count is 500 cells/mm^3^ in adults. The cART status was category into untreated and cART treated.

### Data Analysis

Fifteen participants were excluded from the final analysis because of the insufficient weight of hair samples (less than 20mg) for assaying cortisol and cortisone (n=13) or unavailable data of pain measure (n=2). This left 431 participants for analysis.

Hair cortisol and cortisone levels were not normally distributed as indicated by the Kolmogorov–Smirnov test and were thus Winsorized (5th/95th percentile),^53^ and Box-Cox transformed^54^ for effectively reduced the skewness statistic. Univariate linear regression analysis was used to explore the associations of hair cortisol or hair cortisone with sociodemographic and clinical characteristics. The general linear model was used to examine differences in transformed glucocorticoid levels among the three pain studied groups. Findings of significant main effects were followed up by post hoc comparisons using LSD. Multivariate analysis of covariance was adjusted for sociodemographic and clinical characteristics. All data analyses were performed using SPSS 26.0 (SPSS Inc, Chicago, IL).

## Results

Of the 431participants with a mean (SD) age of 41 (8) years (Table 1), 68% were male, 61.9% were Han ethnicity, 78.9% were married, 77.7% were employed, 91.2% were receiving cART. Most of the sample had low levels of education and income, with 89.6% reporting not complete junior high school and 76.1% reporting a monthly household income of less than 3000 Chinese Yuan (or approximately US$460 during the time of the survey). The median CD4+ T cell count was 359 cells/mm^3^, and the median duration of HIV diagnosis was 1.50 years. Among the 431 PLHIV, 273 (63.3%) reported none pain, 87 (20.2%) reported very mild or maid pain, and 71 (16.5%) reported moderate, severe, or very severe pain. The median cortisol and cortisone levels were 7.60 pg/mg and 44.02 pg/mg, respectively. Hair cortisol and hair cortisone levels were positively correlated (*r* = 0.26, *p* < 0.001).

**Table 1:** Characteristics of the 431 Participants

Characteristics	n(%), M (SD) or Median (IQR)
Age	41.33 (8.38)
Gender	
Male	293 (68.0%)
Female	138 (32.0%)
Ethnicity	
Han	267 (61.9%)
No Han	164 (38.1%)
Marital status	
Married	340 (78.9%)
Other	91(21.1%)
Education	
≤ 9 years	386 (89.6%)
> 9 years	45 (10.4%)
Employment	
Employed	335 (77.7%)
Unemployed	96 (22.3%)
Income level	
≥3000 Yuan per month	103(23.9%)
< 3000 Yuan per month	328 (76.1%)
Years since HIV diagnosis	1.50 (0.92–2.25)
cART status	
Yes	393 (91.2%)
No	38 (8.8%)
CD4 count, cells/mm3	359 (204–532)
Pain severity	
None	273 (63.3%)
Very mild	37 (8.6%)
Mild	50 (11.6%)
Moderate	63 (14.6%)
Severe	5 (1.2%)
Very severe	3 (0.7%)
Hair cortisol, pg/mg	7.60 (4.98–11.74)
Hair cortisone, pg/mg	44.02 (31.12–67.15)

**Abbreviations:** M, mean; SD, standard deviation; IQR, interquartile range; cART, combination antiretroviral therapy.

Table 2 shows the univariate regression of hair cortisol and cortisone levels with sociodemographic and clinical characteristics, respectively. Age, no-Han ethnicity, and be married were positively associated with hair cortisol levels. Female sex was positively associated with both hair cortisol and cortisone levels. Education levels and income levels were negatively associated with hair cortisol levels. Clinical characteristics were not associated with either hair cortisol levels or hair cortisone levels.

**Table 2:** Univariate Regressions of Hair Cortisol and Cortisone Levels with Sociodemographic and Clinical Characteristics

	Hair Cortisol		Hair Cortisone	
	β	p	β	p
Age	0.111	0.022	0.066	0.173
Gender	0.084	0.082	0.214	< 0.001
Ethnicity	0.096	0.045	0.022	0.642
Marital status	0.082	0.088	0.001	0.981
Education level	−0.103	0.033	−0.013	0.792
Employment status	−0.018	0.704	−.0.037	0.444
Monthly income level	−0.191	< 0.001	−0.011	0.825
Years since HIV diagnosis	−0.026	0.597	0.015	0.758
cART status	−0.026	0.584	−0.007	0.889
CD4+ T cell count	0.016	0.741	0.075	0.118

**Abbreviations:** β, standardized coefficient; cART, combination antiretroviral therapy.

**Figure 1:**
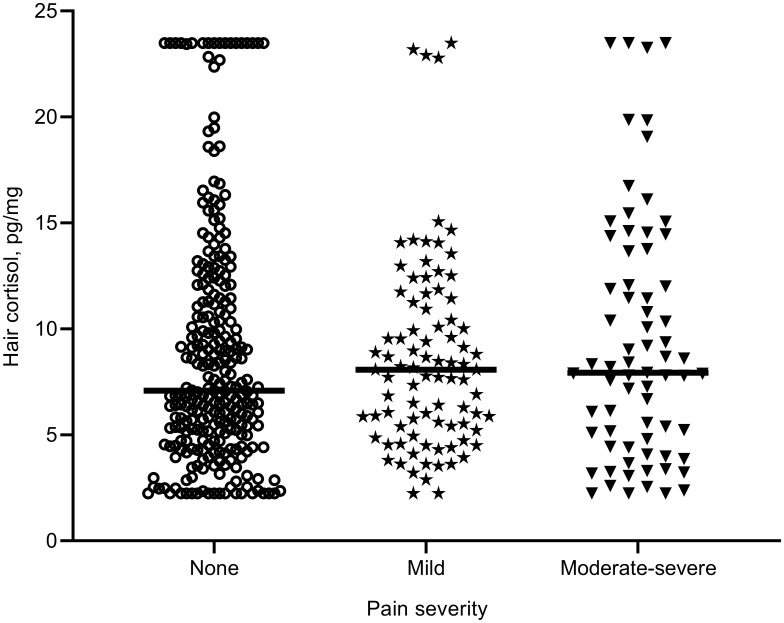
Hair cortisol levels (pg/mg) in none, mild, and moderate-severe pain groups. Horizontal lines represent the group median.

The median hair cortisol levels were 7.09 pg/mg, 8.07 pg/mg, and 7.93 pg/mg for the none, maid, and moderate-severe pain groups, respectively (see Figure 1). No significant difference was found in hair cortisol levels among the three pain groups (*F*=0.14, *p*=0.868).

**Figure 2:**
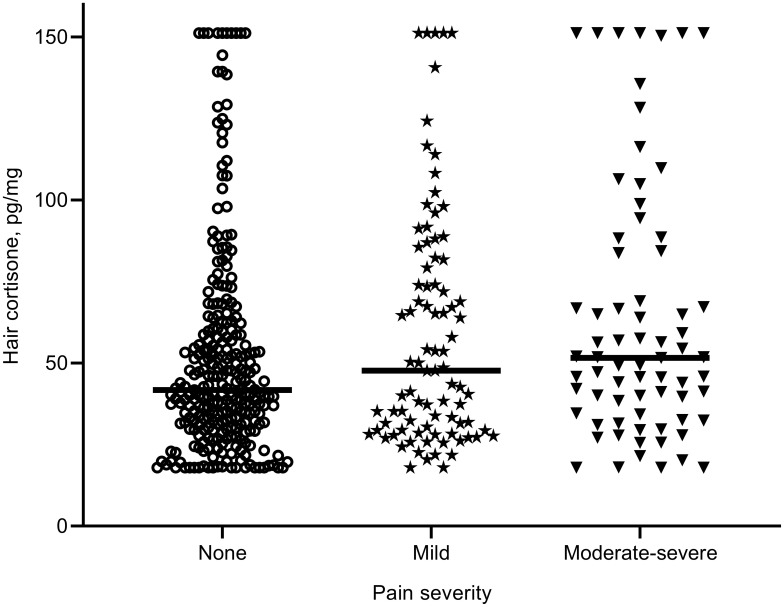
Hair cortisone levels (pg/mg) in none, mild, and moderate-severe pain groups. Horizontal lines represent the group median.

The median hair cortisone levels were 41.78 pg/mg, 47.71 pg/mg, and 51.62 pg/mg for the none, maid, and moderate-severe pain groups, respectively (see Figure 2). A significant difference was detected in hair cortisone levels (*F*=3.90, *p*=0.021). Post hoc analyses for transformed data revealed that PLHIV with moderate-severe pain had higher hair cortisone levels than those with none pain (*p*=0.014) and had marginally higher cortisone levels than those with maid pain (*p*=0.070), with no difference between the latter two groups (*p*>0.10). Adjusting for the covariates on these measures also did not alter the result for hair cortisol levels (*F*=0.02, *p*=0.983) or hair cortisone levels (*F*=4.03, *p*=0.018).

## Discussion

The present study investigated whether pain severity was associated with hair glucocorticoid levels, a novel method of measuring long-term glucocorticoid exposure, among a large cohort of Chinese PLHIV. We found nonsignificant difference in hair cortisol levels among the three pain groups. However, PLHIV with moderate-severe pain had higher hair cortisone levels than those with mild pain or none pain, with no difference between PLHIV with mild pain and none pain.

To the best of our knowledge, only two previous studies investigated the relationship between pain and hair cortisol levels in patients with chronic pain, and no study has directly examined the relationship between pian and hair cortisone levels. Our finding regarding the lack of association of pain severity with hair cortisol levels in PLHIV is inconsistent with the two previous studies that reported higher hair cortisol levels in patients with chronic pain than controls.^21^,^24^ However, we found that PLHIV with moderate-severe pain had higher hair cortisone levels than those with none pain or mild pain. There are two potential explanations for why significant associations were demonstrated for hair cortisone levels, but not hair cortisol levels in our study. Firstly, hair cortisone levels could indicate more systematic glucocorticoid levels than hair cortisol levels. In line with the previous evidence,^39^,^55^,^56^ our data also found that hair cortisone levels were higher than hair cortisol levels (Table 1) and positively correlated. Previous studies also found that salivary cortisone levels demonstrated a stronger correlation with free serum cortisol levels than total serum cortisol levels and salivary cortisol levels^25^ and salivary cortisone levels showed a considerable correlation with hair cortisone levels.^29^ Therefore, hair cortisone levels have been employed as an additional measure of systematic glucocorticoid levels.^26^,^27^ Secondly, hair cortisone levels also appear- to hold certain benefits to hair cortisol levels. For instance, both our study (see Table 2) and previous studies found hair cortisol levels were influenced by additional factors.^51^,^52^ Other studies have also found stronger associations between hair cortisone levels than hair cortisol levels and variables studied, including Parkinson’s disease,^40^ cardiometabolic variables,^37^,^56^ and stress-related variables.^36^,^40^ One study found diagnostic accuracy for Cushing’s syndrome was significantly better for hair cortisone levels than hair cortisol levels.^57^ Therefore, along with previous studies, our study also provides implications for future research to consider both hair cortisol levels and hair cortisone levels, rather than hair cortisol levels alone to represent long-term glucocorticoid levels. Regardless of the relatively little is known about cortisone’s physiological significance, our findings regarding the association between pain severity and hair cortisone levels indicated that health providers in caring for PLHIV should consider interventions (eg, mindfulness) to reduce the long-term glucocorticoid levels and to manage pain.^58^,^59^

We found that clinical characteristics were not associated with hair cortisol or cortisone levels. Two recent studies also reported no association between hair cortisol levels and CD4+ T cell counts^42^ or cART status.^41^ In addition, our results contribute to HIV research by exploring the association of several other sociodemographic variables with hair glucocorticoid levels in a large cohort study and other fields by confirming the association of those variables with hair glucocorticoid levels previously observed in a different population.^51^,^52^

Several study limitations need to be acknowledged. First, the current study was based on cross-sectional data, which prevents making causal inferences. Future research should benefit from using longitudinal designs to investigate whether the change in glucocorticoid levels is consistent in PLHIV who experienced pain and whether the possible cause of pain in PLHIV is abnormalities of the glucocorticoid levels. Second, we only employed single-item questions to assess pain severity. Several other essential pain components were not evaluated in our study, including the pain site, chronicity and frequency of pain, pain coping, and pain medical history. Future research should benefit from employing multiple validity pain scales (eg, brief pain inventory and verbal numerical rating scale) that can include those components of pain. Third, because all participants are from Guangxi, China, these findings may not be generalizable to other PLHIV settings. Fourth, while data were not available in the current study on some other potential factors (eg, physical exercise) that might influence hair glucocorticoid levels,^30^,^60^ those factors should be considered in future research.

## Conclusion

In summary, this study is the first to report that greater pain severity is associated with higher hair cortisol levels among Chinese PLHIV. In order to reduce the long-term glucocorticoid levels, interventions managing pain should be considered for PLHIV with moderate-severe pain. Future work will focus on the longitudinal relationship between these variables and further explore hair cortisone’s utility as a neuroendocrine biomarker in PLHIV.
